# Proactive Coping and Mindfulness are Associated with Less Stress in Older Adults

**DOI:** 10.1093/geroni/igab046.3276

**Published:** 2021-12-17

**Authors:** Lyndsey Graham, Shevaun Neupert

**Affiliations:** North Carolina State University, Raleigh, North Carolina, United States

## Abstract

We examined the consequences of both chronic and life-event stressors for older adults, as well as antecedent strategies, such as proactive coping and mindfulness, that may mitigate stress. Given the potential negative outcomes associated with stress in older adulthood, exploring strategies to reduce or mitigate the negative impact of stress may be useful in promoting well-being in adulthood. Proactive coping involves an accumulation of resources that leads to reduced or avoided stressors in the future (Aspinwall & Taylor, 1997). Mindfulness calls an individual’s attention to the present moment, or may be characterized as an open, accepting attitude (Brown & Ryan, 2003). Using data from the Mindfulness and Anticipatory Coping Everyday study (English et al., 2019; Neupert & Bellingtier, 2017), 296 older adults in the United States, aged 60-90 years (M = 64.67, SD = 4.36), participated in relevant online survey measures. Results from multiple regression analyses suggested that people high in both chronic stress and life event stress had worse health, and that people high in proactive coping and mindfulness reported less stress. Study results underscore the impact of stress on health outcomes, and provide support for the use of antecedent strategies to address negative impacts of stress.

